# Cardiac resynchronization therapy with a defibrillator (CRTd) in failing heart patients with type 2 diabetes mellitus and treated by glucagon-like peptide 1 receptor agonists (GLP-1 RA) therapy vs. conventional hypoglycemic drugs: arrhythmic burden, hospitalizations for heart failure, and CRTd responders rate

**DOI:** 10.1186/s12933-018-0778-9

**Published:** 2018-10-22

**Authors:** Celestino Sardu, Pasquale Paolisso, Cosimo Sacra, Matteo Santamaria, Claudio de Lucia, Antonio Ruocco, Ciro Mauro, Giuseppe Paolisso, Maria Rosaria Rizzo, Michelangela Barbieri, Raffaele Marfella

**Affiliations:** 1 Department of Medical, Surgical, Neurological, Metabolic and Aging Sciences, University of Campania “Luigi Vanvitelli”, Piazza Miraglia, 2, 80138 Naples, Italy; 2 Cardiovascular and Arrhythmias Department, John Paul II Research and Care Foundation, Campobasso, Italy; 3 Center for Translational Medicine, Temple University, Philadelphia, USA; 4 Cardiovascular Diseases Department, Cardarelli Hospital, Naples, Italy

## Abstract

**Objectives:**

To evaluate clinical outcomes in patients with diabetes, treated by cardiac resynchronization therapy with a defibrillator (CRT-d), and glucagon-like peptide 1 receptor agonists (GLP-1 RA) in addition to conventional hypoglycemic therapy vs. CRTd patients under conventional hypoglycemic drugs.

**Background:**

Patients with diabetes treated by CRTd experienced an amelioration of functional New York Association Heart class, reduction of hospital admissions, and mortality, in a percentage about 60%. However, about 40% of CRTd patients with diabetes experience a worse prognosis.

**Materials and methods:**

We investigated the 12-months prognosis of CRTd patients with diabetes, previously treated with hypoglycemic drugs therapy (n 271) vs. a matched cohort of CRTd patients with diabetes treated with GLP-1 RA in addition to conventional hypoglycemic therapy (n 288).

**Results:**

At follow up CRTd patients with diabetes treated by GLP-1 RA therapy vs. CRTd patients with diabetes that did not receive GLP-1 RA therapy, experienced a significant reduction of NYHA class (p value < 0.05), associated to higher values of 6 min walking test (p value < 0.05), and higher rate of CRTd responders (p value < 0.05). GLP-1 RA patients vs. controls at follow up end experienced lower AF events (p value < 0.05), lower VT events (p value < 0.05), lower rate of hospitalization for heart failure worsening (p value < 0.05), and higher rate of CRTd responders (p value < 0.05). To date, GLP-1 RA therapy may predict a reduction of AF events (HR 0.603, CI [0.411–0.884]), VT events (HR 0.964, CI [0.963–0.992]), and hospitalization for heart failure worsening (HR 0.119, CI [0.028–0.508]), and a higher CRT responders rate (HR 3.707, CI [1.226–14.570]).

**Conclusions:**

GLP-1 RA drugs in addition to conventional hypoglycemic therapy may significantly reduce systemic inflammation and circulating BNP levels in CRTd patients with diabetes, leading to a significant improvement of LVEF and of the 6 min walking test, and to a reduction of the arrhythmic burden. Consequently, GLP-1 RA drugs in addition to conventional hypoglycemic therapy may reduce hospital admissions for heart failure worsening, by increasing CRTd responders rate.

*Trial registration* NCT03282136. Registered 9 December 2017 “retrospectively registered”

## Introduction

Type 2 diabetes mellitus (T2DM) is a frequent co-morbidity, and a negative prognostic risk factor in patients with heart failure and reduced left ventricle ejection fraction (HFrEF), [1]. Indeed, T2DM induces a pro-oxidative/inflammatory status, that altering molecular, metabolic, electrical, and mechanical cardiac functions, may consequently lead to HFrEF [1, 2]. In this setting, recently the CHARM trial reported in patients with diabetes a cumulative incidence rate of cardiovascular death or hospitalization for heart failure approximately of 40% over 3 years, with a risk for first hospital admission for heart failure of 155.4 per 1000 patient-years [3]. In this population of HFrEF patients with diabetes the cardiac resynchronization therapy with a defibrillator (CRT-d) is an effective treatment, to improve cardiac performance and functional New York Association Heart (NYHA) class, and to reduce hospital admissions and mortality [4]. To date, these clinical effects are observed in about 60% of all treated patients, named as “CRTd responders” [4]. On the other hand, about the 40% of patients with diabetes show an increasing trend toward heart failure (HF) disease progression, hospital admissions, and deaths, and are defined as “CRTd non responders” [4]. The loss of the CRTd’ therapeutic effect in patients with diabetes may be due to multiple molecular, metabolic, electrical, and mechanical cardiac alterations [4]. Moreover, there is an increasing necessity to find new treatments to ameliorate glucose homeostasis, to control the worsening of T2DM, and to improve clinical outcomes in HFrEF patients with diabetes. In this setting, in last years new hypoglycemic drugs named glucagon-like peptide 1 receptor agonists (GLP-1 RA), have been safety introduced and used also in failing heart patients with diabetes [5]. Intriguingly, GLP-1 RA did not increase the risk of hospitalization for heart failure in patients with diabetes [5]. Actually, at our knowledge there are not studies investigating the effects of GLP-1 RA in addition to conventional hypoglycemic therapy in T2DM failing heart patients treated by CRT-d. Therefore, our study hypothesis was that, GLP-1 RA in addition to conventional hypoglycemic therapy as compared to the conventional hypoglycemic drugs therapy may ameliorate heart function, and clinical outcomes in a population of HFrEF patients with diabetes treated by CRT-d. Moreover, in this study we evaluated the effects of GLP-1 RA in addition to conventional hypoglycemic therapy vs. conventional hypoglycemic drugs therapy in a population of T2DM failing heart patients treated by CRT-d. To date, in this study T2DM patients affected by HFrEF after CRT-d implant were divided randomly in patients receiving GLP1 RA therapy plus conventional hypoglycemic therapy vs. patients under conventional hypoglycemic therapy. In these patients we aimed to investigate at 12 months follow up all cause of deaths, cardiac deaths, hospitalizations for HF worsening, CRT-d responders rate, and the arrhythmic burden: atrial fibrillation (AF) events, ventricular tachycardia (VT) events, ventricular fibrillation (VF) events, internal cardioverter defibrillator (ICD) shocks, and strokes events.

## Materials and methods

### Study population

A base cohort of 579 patients newly treated with non insulin anti-diabetic drugs (metformin, sulfonylureas, thiazolidinediones, glucosidase inhibitors, guar gum, meglitinides, etc.) between 1 January 2010 and 1 January 2017 was assembled. T2DM was diagnosed according to American Diabetes Association criteria [6]. To establish T2DM patients treatment, the screened patients answered a specific questionnaire about medicines used for diabetes treatment, the date of the beginning and end of treatment, route of administration, and duration of use [6]. Therefore, 559 T2DM patients affected by HFrEF were enrolled in the study. The diagnosis of HFrEF was made as indicated by international guidelines on heart failure disease management [2]. After study enrollment phase T2DM patients received a CRT-d treatment according to the international guidelines [2]. Moreover, we randomized the CRTd patients with diabetes under conventional hypoglycemic drug therapy to an GLP1 RA treatment by a computer generating code program. However, 288 CRTd patients with diabetes received GLP-1 RA plus conventional drug therapy, and named as GLP-1 RA group [6]. The remaining patients, 271 CRTd patients with diabetes, did not receive GLP-1 RA therapy, and remained under conventional hypoglycemic drug therapy, and named as conventional group or controls.

Inclusion criteria were: At least 18 years of age, affected by T2DM without insulin anti-diabetic prescription, without GLP1 RA treatment before to receive CRTd, clinical history of stable chronic heart failure, New York Heart Association (NYHA) functional class II or III, left bundle branch block, severe left ventricle ejection fraction reduction (LVEF < 35%), stable sinus rhythm, candidates to receive a CRT-d treatment according to the international guidelines [2].

Exclusion criteria were: Insulin therapy and GLP-1 RA therapy before and at any time of the study, age < 18 or > 75 years, ejection fraction > 35%, previous implant of implantable cardioverter defibrillator (ICD), CRT-d and/or pacemaker, absence of informed patient consent, and any condition that would make survival for 1 year unlikely.

### Study design

This was an observational multicenter, prospective randomized study conducted at University of Campania Luigi Vanvitelli (Naples, Italy), at Cardarelli Hospital (Naples, Italy), at Catholic University of Sacred Heart (Campobasso, Italy), and at John Paul II Research and Care Foundation (Campobasso, Italy). In this study we enrolled a consecutive population of 560 T2DM patients affected by heart failure (HF) with indication to receive a cardiac resynchronization therapy with defibrillator (CRT-d). After CRT-d, 559 consecutive T2DM patients under conventional hypoglycemic drug therapy were divided in two groups as described before in the text. The clinical characteristics of T2DM patients were well matched and balanced between the two groups of study. Before interventions, the baseline laboratory studies, including HbA1c, lipid panel, and fibrinogen, were determined. Responders patients to a CRT-d treatment were defined by evidence of left ventricle (LV) reverse remodeling, 6 min-walk test improvement and Minnesota living with heart failure scale improvement as previously described [2]. Enrolled patients were followed by clinical, instrumental assessment, and device telemetric control (at implant, 10 days, 6, and 12 months after discharge). During these visits and device interrogations, we reported arrhythmic events, shocks interventions, and subsequently CRT-d effect in terms of clinical outcomes, CRT responder rate, and clinical events as deaths, cardiac deaths, and hospitalizations for HF worsening.

### Exposure assessment

As reported before [5, 6], we defined current exposure to an antidiabetic drug as any prescription whose duration plus a 30-day grace period included the index date (period accounted for non adherence and for the drug’s biologic half-life). For all patients (GLP-1 RA group vs. conventional group), current exposure was classified by the use of the following drugs: GLP-1 RA-based drugs, two or more oral antidiabetic drugs used in combination, a single oral antidiabetic drug. Oral antidiabetic drugs used in combination served as our primary reference category, since GLP-1 RA-based drugs are second-line or third-line therapy and are thus used at a similar point in the management of the disease in failing heart patients [2, 6].

### Echocardiographic evaluation

At baseline, and at 6th and 12th month of follow up, a trans-thoracic two-dimensional echocardiograms with M-mode recordings, conventional Doppler, and pulsed-wave tissue Doppler imaging (TDI) measurements was performed in each patient using a Philips iE33 echocardiograph (Eindhoven, The Netherlands). Echocardiographic images were acquired in the parasternal long and short axis views. The LV end-diastolic diameter (LVEDD), end-diastolic volume (LVEDV), end-systolic diameter (LVESD), end-systolic volume (LVESV) were measured, and LVEF was determined with the Simpson method [2]. The amount of mitral regurgitation was calculated as the area of the color-flow Doppler regurgitant jet divided by the area of the left atrium in systole, and described as low (+), moderate (++), moderate-severe (+++), and severe (++++), as previously reported [2, 9]. All echocardiographic studies were performed and analyzed by the same study-independent physicians, blinded to the study protocol. Echocardiographic measurements were systematically averaged in five consecutive samples.

### CRT-d implant

Experienced electrophysiologists in CRT implantation performed the three CRT leads positioning in cardiac chambers, and then connected to the CRT-d generator, as previously described [4]. All CRT-d implant procedures were standardized. Physicians used a multipolar and/or a bipolar LV pacing lead, to reach the target left epicardium vessel, and to have the final LV lead position and pacing configuration, by acceptability of pacing thresholds, absence of diaphragmatic stimulation, and anatomic position (chosen position in the target vessel). The final position of the LV pacing lead was assessed with cine fluoroscopy. Implantation duration was defined as the time between skin incision until suture. We used bipolar LV pacing leads (St Jude Medical, Sylmar, CA, USA; Medtronic, Minneapolis, MN, USA), and quadripolar LV pacing leads (Quartet^®^ model 1458Q and Promote Q^®^, St Jude Medical, Sylmar, CA, USA; Attain Performa^®^ model, Medtronic, Minneapolis, MN, USA), over-the-wire, steroid eluting with a in-line connector. LV pacing leads were connected to an appropriate bipolar CRT-d device (CRT-d bipolar device, St Jude Medical, Sylmar, CA, USA; Medtronic, Minneapolis, MN, USA), and/or to a quadripolar CRT-d device (Quadra Assura CRT-d device, St Jude Medical, Sylmar, CA, USA; Viva^®^ QuadXT and Viva^®^ Quad S cardiac CRT-d, Minneapolis, MN, USA).

### Anthropometrics determination

In these patients we evaluated physical examination, vital signs, and review of adverse events. For each patient we evaluated body mass index (BMI) as the ratio between weight in kg and the height squared [2]. The CRT-d was monitored during follow up, reporting the functionality of the system, all arrhythmic events, and device interventions.

### Laboratory analysis

In all these patients we evaluated, after an overnight fast, the plasma glucose, HbA1c, serum lipids, and B type natriuretic peptide (BNP) by enzymatic assays. In all patients, before intervention, and at follow up, we estimated circulating intact GLP-1, and plasma immunologic active form of GLP-1 [7, 8] by using a specific enzyme-linked immunosorbent assay (ELISA) kit (Active GLP-1 7-36, Epitope). We collected patient’s blood samples in ice-cooled blood collection system for plasma GLP-1 preservation tubes (BD P700), and immediately centrifuged at 2500 rpm for 10 min in refrigerated centrifuge. Samples were stored at − 80 °C. GLP-1 levels (Active GLP-1 7-36, Epitope) measurements were obtained after an overnight fast and after breakfast, as previously reported [6–8]. We defined as post-prandial GLP-1 values the mean of the four GLP-1 evaluations. In these patients at baseline, and during follow up we measured inflammatory markers.

### Inflammatory markers

We evaluated at baseline, and after 12 months follow up, circulating serum levels of pro-inflammatory cytokines (tumor necrosis factor-α, TNF α, interleukin-6, IL6), systemic inflammatory markers (C reactive protein, CRP), and leucocytes and neutrophils count as previously reported [2].

### Study endpoints

As study endpoints, we monitored in all CRTd patients with diabetes, and in the GLP-1 RA group vs. non GLP-1 RA group of patients the cardiac deaths, all cause of deaths, hospitalization rate for HF worsening, strokes, CRT-d responders rate, arrhythmic burden of sustained events, and ICD shocks. Cardiac deaths, all cause of deaths, hospitalization for HF worsening, and stroke events were evaluated during office follow up visits 10 days after clinical discharge, and after 6th and 12th month by the treating physician, by telephonic interviews, hospital admissions, and discharge schedules [4, 9]. During follow up visits, NYHA classification was re-assessed, and patients graded their overall condition as unchanged or slightly, moderately, or markedly worsened, or improved since randomization by global self-assessment [2, 4, 9]. All patients were instructed regularly to assess body weight, occurrence of dyspnea, and any clinical symptom. At each visit patients were asked whether medical events or symptoms suggestive of cardiac arrhythmias occurred, and an ECG, an ECG Holter monitoring and the device interrogations were both performed to detect the presence of asymptomatic arrhythmias. Clinical evaluations included physical examination, vital signs, and review of adverse events. A fasting blood (at least 12 h from last meal) was performed for biochemical peripheral blood assay evaluation at every visit. CRTd responders patients were identified by clinical and instrumental evaluation as reported by authors [2]. In addition, at each clinical follow-up, arrhythmic burden [atrial fibrillation (AF), ventricular tachycardia (VT), and ventricular fibrillation (VF)] and ICD shocks were evaluated by CRTd interrogations and reported for each patient. AF was defined as an arrhythmia originating from atrial chambers, and classified as paroxysmal, and/or not paroxysmal as previously described [4, 9]. VT was defined as arrhythmia originating from ventricular chambers, classified in sustained and/or not sustained by arrhythmic event duration [9]. VF was defined as a fibrillating arrhythmia originating from ventricular chambers, and associated to hemodynamic instability, and cardiac arrest [9]. ICD shocks were defined as high energy interventions by CRT-d device to restore sinus rhythm during at risk of life sustained VT and or VF events [9].

### Ethical committee and clinical trial registration

The study was conducted in accordance with the Declaration of Helsinki. The Ethics Committees of all participating institutions approved the protocol. All patients were informed about the study nature, and gave their written informed, and signed consent to participate in the study. The study was registered in ClinicalTrials.gov, clinical Trial Number NCT03282136.

### Statistical methods

A qualified statistician analyzed all collected data. The CRT-d and T2DM patients were divided in GLP-1 RA group of patients vs. non GLP-1 RA group of patients (conventional group or controls), and during follow up visits, and controls in CRT-d responders vs. CRT-d non-responders. We postulated that, the number of patients with alterations in primary and secondary endpoints was significantly different between GLP-1 RA group of patients vs. non GLP-1 RA group of patients. Safety analyses were performed on data from all enrolled patients. Continuous variables were expressed as means and standard deviations, and were tested by two-tailed Student t test for paired or unpaired data, as appropriate, or by one-way analysis of variance (ANOVA) for more than two independent groups of data. The categorical variables were compared by Chi square or Fisher exact test where appropriate. Survival analysis was performed with the use of the Kaplan–Meier method. Predictors of the study endpoints were evaluated by using Cox regression models in patients with GLP-1 RA based drugs as compared with oral antidiabetic-drug combinations. A univariate analysis was conducted to examine the association between single principal clinic, echocardiographic, electrocardiographic characteristics, and GLP-1 RA therapy, and 12 months study outcomes. All variables with p value of less than 0.2 in the univariate analysis were subsequently entered into a multivariate model. In the multivariate model, variables were separately selected and a p value of less than 0.05 was considered significant. For all independent predictors, 95% confidence intervals were calculated. Statistical significance was considered for a p value of less than 0.05. The statistical analysis was performed using the SPSS software package for Windows 17.0 (SPSS Inc., Chicago Illinois). We calculated a sample size with 300 participants for each group, with estimated 80% power to detect a change of 0.015 between the mean MPI of the placebo-treated and actively treated groups, at a 5% level of significance. A 20% Loss due to early withdrawals and/or non-evaluable measurements was assumed and, combined with the effect of stratification on analysis, resulted in the requirement to recruit at last 240 patients per treatment group.

## Results

**Fig. 1:**
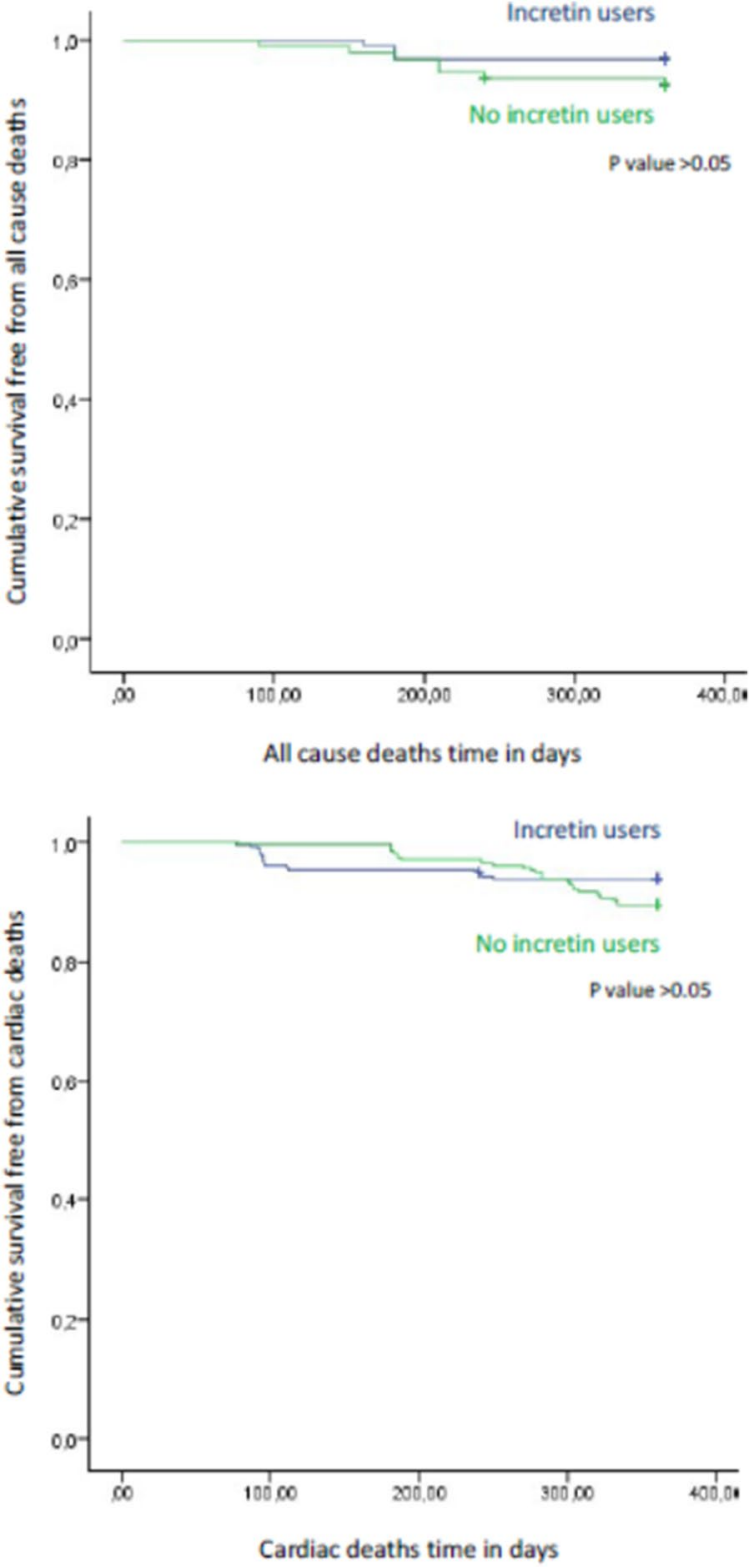
Kaplan survival curve free for all cause deaths (upper part), and cardiac deaths (lower part) event in GLP-1 agonist users (blu color), and non GLP-1 agonist users (green color). p value > 0.05

**Fig. 2:**
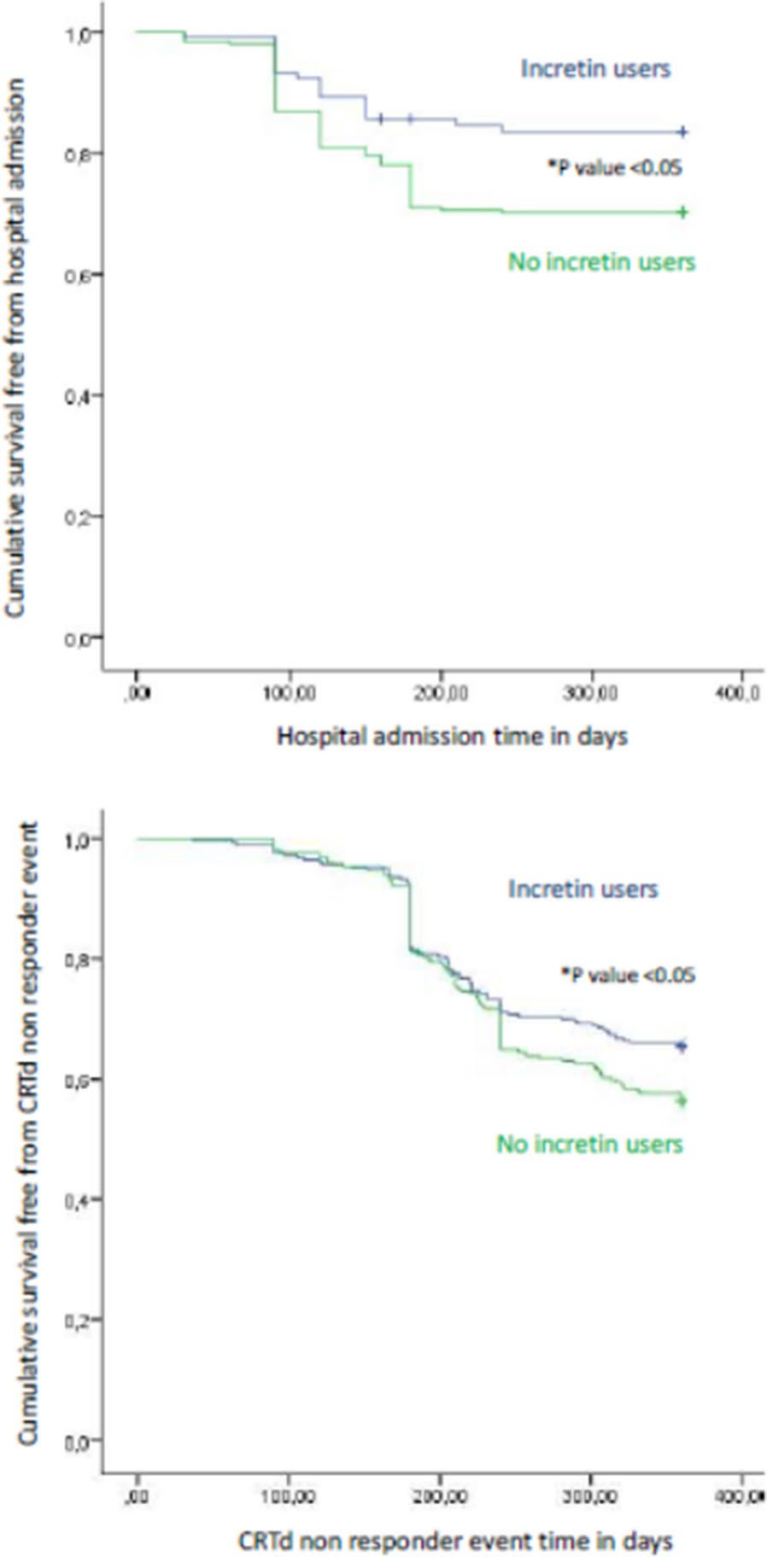
Kaplan survival curve free for hospital admission (upper part), and cardiac resynchronization with a defibrillator (CRTd) response (lower part) event in GLP-1 agonist users (blu color), and non GLP-1 agonist users (green color). *p value < 0.05 for both images

**Fig. 3:**
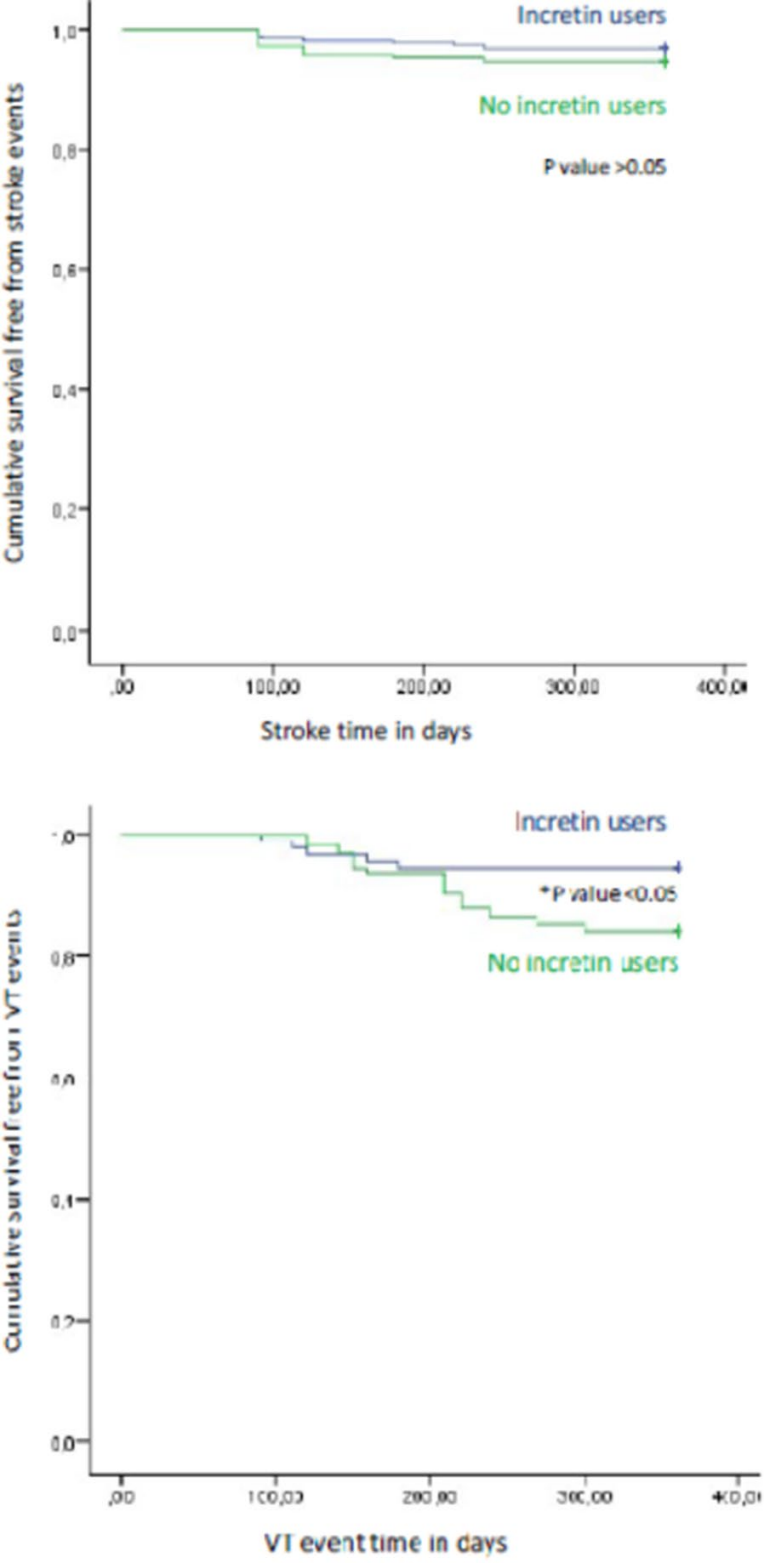
Kaplan survival curve free for stroke events (upper part), and ventricular tachycardia (VT) events (lower part) event in GLP-1 agonist users (blu color), and non GLP-1 agonist users (green color). *p value < 0.05 for VT events

**Fig. 4:**
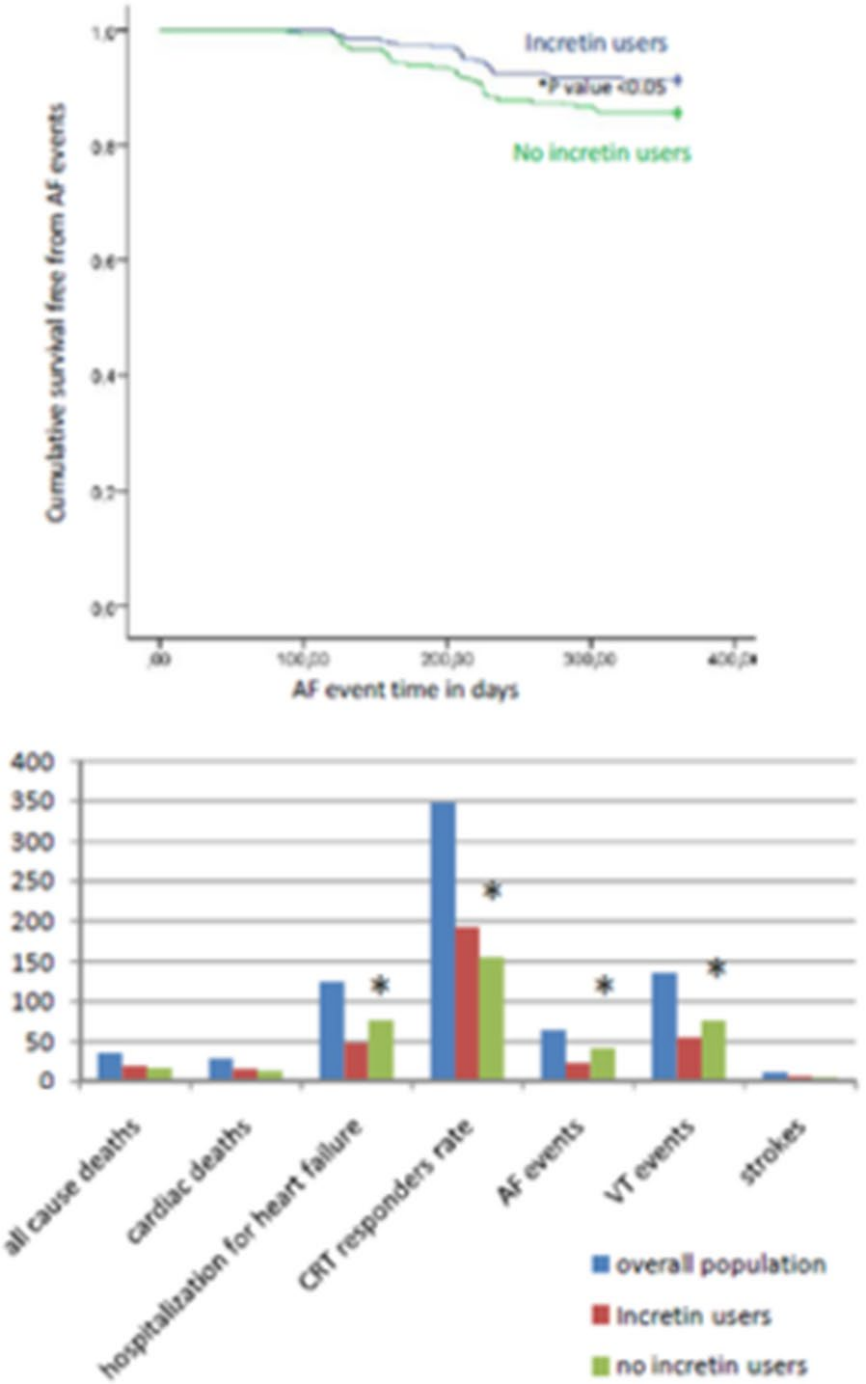
In the upper part of figure the Kaplan survival curve free from atrial fibrillation (AF) events. In lower part the study endpoints events at 12th month of follow up in overall population, patients with diabetes GLP-1 agonist users vs. never GLP-1 agonist users. *p value < 0.05 for both images. AF: atrial fibrillation; CRTd: cardiac resynchronization with a defibrillator; VT: ventricular tachycardia

Enrolled patients with diabetes were 559, divided in GLP-1 RA therapy group (n 288), and non GLP-1 RA therapy group (n 271). Characteristics of study population at baseline were reported in Table 1. At 6th and 12th month of follow up, patients in GLP-1 RA therapy vs. controls experienced a significant increment in postprandial GLP-1 values (19.7 ± 2.4 vs. 11.5 ± 2.3, p value < 0.05, and 19.9 ± 2.5 vs. 11.5 ± 2.3 pmol/L, p value < 0.05). During 6th and 12th month of follow up, GLP-1 RA therapy group vs. controls experienced a significant reduction of NYHA class, associated to higher values of 6 min walking test (6MWT, 309.7 ± 24.6 vs. 226.9 ± 26.7, and 311.5 ± 25.2 vs. 228.2 ± 26.5, p value < 0.05), and to a higher rate of CRTd responders (193 (67%) vs. 155 (57.2%), p value < 0.05 (Table 2a, b). Similarly, comparing GLP-1 RA therapy patients vs. controls there was a significant reduction at 6th and 12th month of follow up of BNP (153.58 ± 12.64 vs. 271.43 ± 13.7, and 146.38 ± 14.14 vs. 262.22 ± 12.95 pg/mL, p value < 0.05), CRP (7.25 ± 0.69 vs. 8.66 ± 0. 94, and 7.23 ± 0.57 vs. 8.32 ± 0. 87 mg/L, p value < 0.05), IL6 (5.53 ± 0.02 vs. 6.24 ± 0.04 pg/mL, and 5.49 ± 0.02 vs. 6.32 ± 0.04 pg/mL, p value < 0.05), and TNFa values (5.36 ± 0.02 vs. 6.32 ± 0.02 pg/mL, and 5.34 ± 0.02 vs. 6.28 ± 0.02 pg/mL, p value < 0.05) (Table 2a, b). Regard the clinical study outcomes, GLP-1 RA vs. controls patients at follow up end experienced lower rate of hospitalization for heart failure worsening [48 (16.7%) vs. 76 (28.0%), p value < 0.05], higher rate of CRTd responders [193 (67%) vs. 155 (57.2%)], associated to lower number of AF events (23 vs. 41, p value < 0.05), and number of VT events (55 vs. 75, p value < 0.05) (Table 3, Figs. 1, 2, 3 and 4). In addition, in CRTd patients with diabetes the GLP-1 RA therapy vs. controls reduced the number of ATP events (37 vs. 68, p value < 0.05), the number of ICD shocks (9 vs. 43, p value < 0.05) and the number of inappropriate therapy events (12 vs. 21, p value < 0.05), with an increased number of appropriate therapy events (74 vs. 38, p value < 0.05). Table 3. Finally, 35 patients [19 (6.6%) GLP-1 RA treated patients vs. 16 (5.9%) controls, p value > 0.05] experienced all cause of deaths, and that 28 patients [15 (5.2%) GLP-1 RA patients vs. 13 (4.8%) controls, p value > 0.05] experienced cardiac deaths (Table 3).

**Table 1:** Clinical characteristics of study population as overall patients (n 559), and GLP-1 agonist therapy (n 288) vs. no-GLP-1 agonist therapy patients (n 271) at baseline

Parameters	Overall population (n 559)	GLP-1 receptor agonist therapy (n 288)	No GLP-1 agonist therapy (n 271)	p value
Age	72 ± 6	72 ± 7	72 ± 6	–
Male (%)	403 (72.1)	206 (71.5)	197 (72.6)	–
Smokers (%)	108 (52.4)	49 (49.5)	59 (55.1)	–
Hypertension (%)	394 (70.5)	200 (69.4)	194 (71.6)	–
Dyslipidemia (%)	193 (34.5)	101 (35.1)	92 (33.9)	–
Plasma glucose (mg/dL)	197.4 ± 21.4	197.4 ± 24.6	197.8 ± 23.2	–
HbA1c (mmol/mol)	58.1 ± 16.1	58.2 ± 16.2	58.0 ± 16.0	–
Basal GLP-1 (pmol/L)	6.29 ± 0.65	5.86 ± 0.68	5.82 ± 0.62	–
Postprandial GLP-1 (pmol/L)	14.49 ± 2.79	13.17 ± 2.76	12.5 ± 2.83	–
BMI > 30 kg/m^2^ (%)	35 (6.3)	18 (6.2)	17 (6.3)	–
COPD (%)	96 (17.2)	50 (17.4)	46 (17)	–
Renal disease (%)	107 (19.1)	56 (19.4)	51 (18.9)	–
Ischemic heart failure (%)	381 (68%)	195 (67.7)	186 (68.6)	–
II NYHA class (%)	133 (23.8)	67 (23.3)	66 (24.4)	–
III NYHA class (%)	426 (76.2)	221 (76.7)	205 (75.6)	–
QRS duration (ms)	138.5 ± 9.4	137.8 ± 9.2	139.2 ± 9.6	–
6MWT	187.90 ± 26.10	188.82 ± 26.45	186.93 ± 25.74	–
CRTd bipolar pacing (%)	121 (21.6)	65 (22.6)	56 (20.6)	–
CRTd multipolar pacing (%)	438 (78.4)	227 (78.8)	211 (77.8)	–
Echocardiographic parameters		288	271	
LVEF (%)	27 ± 5	27 ± 6	28 ± 4	–
LVEDd (mm)	67 ± 8	69 ± 6	66 ± 9	–
LVESd (mm)	43 ± 7	42 ± 6	44 ± 8	–
LVEDv (mL)	197 ± 39	194 ± 29	200 ± 48	–
LVESv (mL)	135 ± 28	133 ± 21	138 ± 35	–
Mitral insufficiency				
+ (%)	280 (50.1)	131 (45.5)	133 (49.2)	–
++ (%)	217 (38.8)	110 (38.2)	106 (39.1)	–
+++ (%)	62 (11.1)	47 (16.3)	32 (11.8)	–
Medications at baseline				
Amiodarone (%)	117 (20.9)	60 (20.8)	57 (21)	–
Aspirin (%)	225 (40.2)	119 (41.3)	106 (39.1)	–
ACE inhibitors (%)	152 (27.2)	132 (45.8)	120 (44.2)	–
ARS blockers (%)	168 (30)	85 (29.5)	83 (30.6)	–
Sacubitril/valsartan (%)	140 (25)	73 (25.3)	67 (24.7)	–
Beta blockers				
Carvedilol (%)	171 (30.6)	89 (30.9)	82 (30.3)	–
Bisoprolol (%)	82 (39.8)	37 (37.4)	45 (42)	–
Warfarin (%)	196 (35.1)	97 (33.7)	99 (36.5)	–
NOAC (%)	111 (19.9)	57 (19.8)	54 (19.9)	–
Tiklopidine (%)	10 (1.8)	5 (1.7)	5 (1.8)	–
Calcium antagonist (%)	19 (3.4)	11 (3.8)	8 (3.0)	–
Ivabradine (%)	165 (29.5)	89 (30.9)	76 (28)	–
Digoxin (%)	176 (31.5)	87 (30.2)	89 (32.8)	–
Loop diuretics (%)	506 (90.5)	265 (92)	241 (88.9)	–
Aldosterone blockers (%)	361 (64.6)	180 (62.5)	181 (66.8)	–
Statins (%)	397 (71)	202 (70.1)	195 (72)	–
Anti diabetic drugs, n (%)				
Insulins (%)	63 (11.3)	33 (11.5)	30 (11.1)	–
Metformin (%)	175 (31.3)	87 (30.2)	90 (33.2)	–
Sulfonylureas (%)	109 (19.5)	56 (19.4)	53 (19.6)	–
Thiazolidinediones (%)	41 (7.3)	22 (7.6)	19 (7.1)	–
GLP-1 agonist (%)	288 (48.1)	288 (100)	–	–
GLP1 agonist (%)				
Liraglutide	71 (12)	68 (23.6)	–	–
Lixenatide	117 (21)	220 (76.4)	–	–
DPP4 inhibitors (%)				
Sitagliptin	–	–	–	–
Linagliptin	–	–	–	–
Biomarkers				
Lymphocytes	7.92 ± 2.13	7.92 ± 2.12	7.62 ± 2.36	–
Neutrophiles	5.31 ± 1.81	5.32 ± 1.80	5.26 ± 2.07	–
BNP (pg/mL)	365.5 ± 9.98	353.71 ± 13.45	378.03 ± 14.8	–
CRP (mg/L)	9.39 ± 0.51	9.43 ± 0. 57	9.14 ± 0.56	–
IL6 (pg/mL)	6.65 ± 0.03	6.58 ± 0.02	6.74 ± 0.05	–
TNFa (pg/mL)	6.37 ± 0.01	6.36 ± 0.02	6.37 ± 0.02	–

HbA1c: glicated hemoglobin type A1c; GLP-1: glucagone like peptide 1; BMI: body mass index; COPD: chronic obstructive pulmonary disease; NYHA: New York Heart Association; 6MWT: 6 min walking test; n.s.: not statistical significant; LVEF: left ventricle ejection fraction; LVEDd: left ventricle end diastolic diameter; LVESd: left ventricle end systolic diameter; LVEDv left ventricle end diastolic volume; LVESv: left ventricle end sistolic volume; mitral insufficiency +: low grade; ++: moderate; +++: more than moderate; ACE: angiotensin converting enzyme; ARS: angiotensin receptor; NOAC: new oral anticoagulant; DPP4: Di-Peptidil-Peptidasi IV; BNP: B type natriuretic peptide; CRP: C reactive protein; IL6: interleukine 6; TNFa: tumor necrosis factor alpha. Symbol “–” is for p value > 0.05

**Table 2:** Follow up 6 (a), and 12 months (b)

Parameters	GLP-1 agonist therapy (n 288)	No GLP-1 agonist therapy (n 271)	p value
(a) 6 months follow up			
Plasma glucose (mg/dL)	189.2 ± 16.4	189.6 ± 15.1	n.s.
HbA1c (mmol/mol)	54.1 ± 12.3	54.0 ± 12.1	n.s
Basal GLP-1 (pmol/L)	6.84 ± 0.71	6.02 ± 0.65	< 0.05*
Postprandial GLP-1 (pmol/L)	19.7 ± 2.4	11.5 ± 2.3	< 0.05*
I NYHA class	17 (5.9)	7 (2.6)	< 0.05*
II NYHA class	117 (40.6)	79 (29.1)	< 0.05*
III NYHA class	143 (49.6)	172 (63.5)	< 0.05*
IV NYHA class	11 (3.8)	13 (4.8)	n.s
QRS duration	121.8 ± 9.8	123.5 ± 9.4	n.s.
6MWT	309.7 ± 24.6	226.9 ± 26.7	< 0.05*
Echocardiographic parameters			
LVEF (%)	32 ± 8	28 ± 6	< 0.05*
LVEDd (mm)	66 ± 4	63 ± 7	n.s.
LVESd (mm)	36 ± 4	38 ± 5	n.s.
LVEDv (mL)	165 ± 24	170 ± 40	n.s.
LVESv (mL)	111 ± 16	119 ± 32	< 0.05*
Mitral insufficiency			
+ (%)	141 (48.9)	135 (49.8)	n.s.
++ (%)	114 (39.6)	101 (37.3)	< 0.05*
+++ (%)	33 (11.5)	35 (12.9)	n.s.
CRTd responders (%)	193 (67.4)	155 (57.2)	< 0.05*
Biomarkers			
Lymphocytes	7.89 ± 2.17	7.52 ± 2.39	n.s.
Neutrophiles	5.37 ± 1.82	5.67 ± 2.12	n.s.
BNP (pg/mL)	153.58 ± 12.64	271.43 ± 13.7	< 0.05*
CRP (mg/L)	7.25 ± 0.69	8.66 ± 0. 94	< 0.05*
IL6 (pg/mL)	5.53 ± 0.02	6.24 ± 0.04	< 0.05*
TNFa (pg/mL)	5.36 ± 0.02	6.32 ± 0.02	< 0.05*
(b) 12 months follow up			
Plasma glucose (mg/dL)	185.3 ± 15.8	186.6 ± 15.5	n.s.
HbA1c (mmol/mol)	52.7 ± 12.5	53.1 ± 12.4	n.s.
Basal GLP-1 (pmol/L)	6.76 ± 0.74	6.02 ± 0.67	n.s.
Postprandial GLP-1 (pmol/L)	19.9 ± 2.5	11.5 ± 2.3	< 0.05*
I NYHA class	17 (5.9)	7 (2.6)	< 0.05*
II NYHA class	115 (39.9)	80 (29.5)	< 0.05*
III NYHA class	144 (50)	170 (62.7)	< 0.05*
IV NYHA class	12 (4.2)	14 (5.2)	n.s
QRS duration	121.8 ± 9.8	123.5 ± 9.4	n.s.
6MWT	311.5 ± 25.2	228.2 ± 26.5	< 0.05*
Echocardiographic parameters			
LVEF (%)	32 ± 8	28 ± 6	< 0.05*
LVEDd (mm)	66 ± 4	63 ± 7	n.s.
LVESd (mm)	36 ± 4	38 ± 5	n.s.
LVEDv (mL)	165 ± 24	170 ± 40	n.s.
LVESv (mL)	111 ± 16	119 ± 32	< 0.05*
Mitral insufficiency			
+ (%)	144 (50)	137 (50.6)	n.s.
++ (%)	116 (40.3)	102 (37.6)	< 0.05*
+++ (%)	28 (9.7)	32 (11.8)	n.s.
CRTd responders (%)	193 (67.4)	155 (57.2)	< 0.05*
Biomarkers			
Lymphocytes	7.90 ± 2.14	7.66 ± 2.36	n.s.
Neutrophiles	5.35 ± 1.81	5.56 ± 2.09	n.s.
BNP (pg/mL)	146.38 ± 14.14	262.22 ± 12.95	< 0.05*
CRP (mg/L)	7.23 ± 0.57	8.32 ± 0. 87	< 0.05*
IL6 (pg/mL)	5.49 ± 0.02	6.32 ± 0.04	< 0.05*
TNFa (pg/mL)	5.34 ± 0.02	6.28 ± 0.02	< 0.05*

Clinical characteristics of study population as overall patients (n 559), and GLP-1 agonist therapy (n 288) vs. no-GLP-1 agonist therapy patients (n 271) at 6th and 12th month of follow up

HbA1c: glicated hemoglobin type A1c; GLP-1: glucagone like peptide 1; NYHA: New York Heart Association; 6MWT: 6 min walking test; CRTd: cardiac resynchronization with a defibrillator; BNP: B type natriuretic peptide; IL6: interleukine 6; LVEF: left ventricle ejection fraction; LVEDd: left ventricle end diastolic diameter; LVESd: left ventricle end systolic diameter; LVEDv left ventricle end diastolic volume; LVESv: left ventricle end sistolic volume; mitral insufficiency +: low grade; ++: moderate; +++: more than moderate; TNFa: tumor necrosis factor alpha. n.s.: not statistical significant

p value < 0.05 is statistical significant, and marked as *

**Table 3:** Clinical outcomes at 12th month in GLP-1 agonist users vs. never-GLP-1 agonist users

Study outcomes	Overall population (n 559)	GLP-1 agonist therapy (n 288)	No GLP-1 agonist therapy (n 271)	p value
All cause deaths (%)	35 (6.3)	19 (6.6)	16 (5.9)	n.s.
Cardiac deaths (%)	28 (5.0)	15 (5.2)	13 (4.8)	n.s.
Hospitalization for heart failure (%)	124 (22.2)	48 (16.7)	76 (28.0)	< 0.05*
CRTd responders rate (%)	348 (62.2)	193 (67)	155 (57.2)	< 0.05*
AF events n of events	64	23	41	< 0.05*
VT events n of events	135	55	75	< 0.05*
Stroke (%)	11 (2)	6 (2.1)	5 (1.8)	n.s
ATP n of events	105	37	68	< 0.05*
CRTd shocks n of events	52	9	43	< 0.05*
Appropriate therapy n of events	112	74	38	< 0.05*
Inappropriate therapy n of events	43	12	21	< 0.05*

AF: atrial fibrillation; VT: ventricular tachycardia; CRTd: cardiac resynchronization with a defibrillator; n: number

At multivariate Cox regression analysis, BNP value (HR 1.120, [1.001–1.401] CI 95%, p value 0.017), and the GLP-1 RA therapy (HR 0.119, [0.028–0.508] CI 95%, p value 0.004) were predictive of hospitalization for heart failure worsening (Table 4). GLP-1 RA therapy in addition to standard hypoglycemic drugs was predictive of CRT responders rate (HR 3.707 [1.226–14.570], CI 95%, p value 0.026), of AF events (HR 0.603 [0.411–0.884], CI 95%, p value 0.010), and of VT events (HR 0.964 [0.963–0.992], CI 95%, p value 0.012) (Table 4). About VT events, they are predicted also by LVEF (HR 1.160 [1.012–1.290] CI 95%, p value 0.047), and by QRS duration (HR 1.511 [1.160–1.959] CI 95%, p value 0.026) (Table 4) Similarly, LVEF (HR 1.593 [1.122–1.986] CI 95%, p value 0.006), BNP (HR 1.101 [1.001–1.210] CI 95%, p value 0.017), and QRS duration (HR 1.182 [1.012–1.552] CI 95%, p value 0.043) may predict AF events at 12 months of follow up (Table 4).

**Table 4:** Cox regression analysis for study endpoints

	Univariate analysis		Multivariate analysis	
	HR (95% CI)	*p value*	HR (95% CI)	*p value*
a. Multivariate cox regression analysis for parameters associated with all cause deaths				
LVEF	1.060 [0.978–1.148]	0.157	0.994 [0.608–1.625]	0.981
6MWT	0.989 [0.974–1.003]	0.131	0.975 [0.869–1.093]	0.661
BNP	1.001 [0.999–1.020]	0.233	0.989 [0.963–1.016]	0.463
TNF alpha	1.666 [0.650–4.273]	0.288	2.253 [0.001–2.671]	0.939
GLP-1	2.980 [0.904–9.824]	0.073	5.127 [0.002–12.548]	0.445
CRP	0.977 [0.933–1.024]	0.336	0.867 [0.002–12.548]	0.548
IL-6	1.254 [0.811–1.939]	0.308	0.004 [0.545–1.380]	0.961
GLP-1 agonist	2.373 [1.087–5.182]	0.030	7.619 [0.004–14.031]	0.597
Age	1.140 [1.082–1.201]	0.001	1.063 [0.805–1.404]	0.665
Obesity	0.045 [0.001–2.916]	0.347	1.139 [0.001–3.434]	0.945
NYHA 3	1.518 [0.731–3.151]	0.263	0.037 [0.001–1.358]	0.273
COPD	3.793 [1.827–7.874]	0.001	0.107 [0.001–1.297]	0.529
QRS duration	1.005 [0.967–1.044]	0.810	0.981 [0.608–1.128]	0.081
Hypertension	1.686 [0.689–4.125]	0.252	0.005 [0.001–2.492]	0.087
Dyslipidemia	0.957 [0.391–2.341]	0.923	0.872 [0.401–4.951]	0.331
Renal dysfunction	1.054 [0.320–3.476]	0.931	3.854 [0.015–8.201]	0.095
Hb1Ac	0.756 [0.435–1.347]	0.354	0.416 [0.072–2.415]	0.328
b. Multivariate cox regression analysis for parameters associated with cardiac deaths				
LVEF	0.952 [0.902–1.006]	0.080	0.438 [0.001–8.345]	0.831
6MWT	0.998 [0.987–1.009]	0.733	1.141 [0.778–1.673]	0.510
BNP	1.001 [0.989–1.010]	0.853	0.993 [0.935–1.054]	0.831
TNF alpha	2.277 [1.091–4.752]	0.028	0.201 [0.011–8.955]	0.727
GLP-1	2.980 [0.904–9.824]	0.073	5.127 [0.002–12.548]	0.445
CRP	0.961 [0.920–1.004]	0.075	4.282 [0.035–5.287]	0.554
IL-6	1.364 [0.976–1.906]	0.069	7.623 [0.002–12.782]	0.724
GLP-1 agonist	1.691 [0.929–3.087]	0.085	0.001 [0.015–1.491]	0.687
Age	1.307 [1.237–1.381]	0.001	2.680 [0.562–12.791]	0.095
Obesity	1.018 [0.316–3.282]	0.976	0.013 [0.005–6.410]	0.772
NYHA 3	8.791 [3.474–22.248]	0.001	0.010 [0.001–7.279	0.633
COPD	2.041 [1.143–3.646]	0.016	0.421 [0.001–1.760]	0.863
QRS duration	0.990 [0.959–1.021]	0.528	2.797 [0.333–23.510]	0.344
Hypertension	1.744 [0.842–3.614]	0.135	0.031 [0.001–2.310]	0.868
Dyslipidemia	3.473 [1.078–11.195]	0.037	4.939 [0.001–12.181]	0.358
Renal dysfunction	0.636 [0.197–2.051]	0.449	0.052 [0.003–1.193]	0.422
Hb1Ac	1.429 [0.911–2.241]	0.120	3.119 [0.384–4.091]	0.167
c. Multivariate cox regression analysis for parameters associated with hospitalization for heart failure				
LVEF	0.978 [0.945–1.012]	0.199	0.927 [0.826–1.041]	0.202
6MWT	1.003 [0.997–1.010]	0.301	1.013 [0.992–1.034]	0.226
BNP	1.002 [1.001–1.020]	0.001	*1.120 [1.001–1.401]*	0.017*
TNF alpha	1.078 [0.665–1.749]	0.761	0.028 [0.000–1.673]	0.420
GLP-1	1.719 [1.077–2.744]	0.023	1.143 [0.451–2.900]	0.778
CRP	0.987 [0.967–1.007]	0.207	0.981 [0.923–1.042]	0.530
IL-6	1.163 [0.926–1.460]	0.193	1.879 [0.017–2.129]	0.272
GLP-1 agonist	1.914 [1.335–2.744]	0.001	*0.119 [0.028–0.508]*	0.004*
Age	1.005 [0.979–1.033]	0.691	0.977 [0.891–1.072]	0.624
Obesity	0.997 [0.488–2.039]	0.994	0.923 [0.826–1.742]	0.983
NYHA 3	1.680 [1.177–2.397]	0.004	1.43 [0.398–1.041]	0.562
COPD	1.126 [0.776–1.633]	0.533	6.870 [0.639–7.383]	0.112
QRS duration	1.005 [0.987–1.024]	0.578	1.031 [0.963–1.102]	0.383
Hypertension	0.875 [0.606–1.265]	0.478	1.381 [0.342–5.583]	0.651
Dyslipidemia	1.932 [1.128–3.311]	0.016	0.354 [0.081–1.541]	0.166
Renal dysfunction	0.889 [0.479–1.649]	0.709	0.565 [0.103–3.098]	0.510
Hb1Ac	1.255 [0.955–1.649]	0.103	0.967 [0.626–1.493]	0.879
d. Multivariate cox regression analysis for parameters associated with CRT responders				
LVEF	1.043 [1.019–1.068]	0.001	1.032 [0.979–1.089]	0.239
6MWT	1.010 [0.996–1.410]	0.897	0.998 [0.987–1.008]	0.665
BNP	1.001 [0.989–1.001]	0.487	1.012 [0.989–1.189]	0.962
TNF alpha	0.950 [0.688–1.312]	0.755	0.508 [0.007–3.712]	0.757
GLP-1	0.840 [0.660–1.069]	0.156	0.757 [0.510–1.123]	0.166
CRP	0.997 [0.986–1.008]	0.601	0.997 [0.966–1.029]	0.851
IL-6	1.007 [0.857–1.183]	0.930	0.823 [0.010–6.662]	0.953
GLP-1 agonist	1.041 [0.841–1.288]	0.714	*3.707 [1.226–14.570]*	0.026*
Age	0.988 [0.972–1.005]	0.154	1.010 [0.957–1.045]	0.992
Obesity	1.394 [0.934–2.080]	0.104	0.564 [0.192–1.656]	0.297
NYHA 3	0.711 [0.573–0.881]	0.020	1.222 [0.646–2.313]	0.538
COPD	0.796 [0.624–1.015]	0.066	2.474 [0.965–6.343]	0.059
QRS duration	0.989 [0.978–1.001]	0.053	0.999 [0.971–1.027]	0.059
Hypertension	1.073 [0.850–1.355]	0.554	0.774 [0.430–1.394]	0.394
Dyslipidemia	0.848 [0.653–1.102]	0.218	1.135 [0.632–2.039]	0.671
Renal dysfunction	0.937 [0.651–1.349]	0.727	1.685 [0.666–4.260]	0.270
Hb1Ac	1.028 [0.864–1.222]	0.756	1.170 [0.949–1.442]	0.141
e. Multivariate cox regression analysis for parameters associated with strokes				
LVEF	0.931 [0.866–1.002]	0.055	0.449 [0.033–7.319]	0.758
6MWT	0.989 [0.973–1.006]	0.203	1.065 [0.232–4.894]	0.935
BNP	1.001 [0.989–1.020]	0.297	0.991 [0.809–1.214]	0.932
TNF alpha	1.062 [0.347–3.250]	0.917	0.016 [0.001–6.694]	0.988
GLP-1	2.315 [0.69–7.761]	0.174	0.020 [0.001–7.072]	0.862
CRP	0.983 [0.935–1.033]	0.502	1.215 [0.002–7.735]	0.953
IL-6	0.617 [0.273–1.394]	0.245	2.159 [0.001–3.829]	0.991
GLP-1 agonist	1.691 [0.740–3.864]	0.213	1.320 [0.001–6.473]	0.913
Age	0.946 [0.885–1.012]	0.104	1.263 [0.004–4.136]	0.937
Obesity	5.241 [2.080–13.205]	0.001	0.031 [0.001–2.678]	0.771
NYHA 3	0.994 [0.447–2.213]	0.989	0.129 [0.001–5.426]	0.960
COPD	1.764 [0.784–3.972]	0.170	0.212 [0.001–1.878]	0.896
QRS duration	1.001 [0.960–1.045]	0.951	1.315 [0.059–2.940]	0.960
Hypertension	1.264 [0.502–3.185]	0.619	2.066 [0.001–11.954]	0.982
Dyslipidemia	0.699 [0.277–1.762]	0.448	0.948 [0.001–3.020]	0.794
Renal dysfunction	0.043 [0.001–16.543]	0.301	0.880 [0.001–1.003]	0.880
Hb1Ac	1.115 [0.412–3.021]	0.830	4.248 [0.001–5.346]	0.752
f. Multivariate cox regression analysis for parameters associated with VT events				
LVEF	1.041 [0.985–1.100]	0.157	*1.160 [1.012–1.290]*	0.047*
6MWT	0.993 [0.983–1.040]	0.203	0.993 [0.983–1.004]	0.194
BNP	1.001 [0.909–1.020]	0.321	1.001 [0.098–1.002]	0.505
TNF alpha	1.475 [0.749–2.904]	0.261	1.009 [0.484–2.101]	0.981
GLP-1	1.839 [0.904–3.739]	0.093	0.878 [0.585–1.317]	0.529
CRP	0.973 [0.940–1.007]	0.121	0.991 [0.958–1.025]	0.609
IL-6	1.384 [1.034–1.853]	0.029	1.076 [0.780–1.485]	0.654
GLP-1 agonist	0.332 [0.185–0.597]	0.001	*0.964 [0.963–0.992]*	0.012*
Age	1.039 [1.001–1.079]	0.046	1.051 [0.816–1.599]	0.066
Obesity	5.478 [3.005–9.987]	0.001	0.174 [0.016–1.599]	0.072
NYHA 3	2.773 [1.562–4.923]	0.001	0.541 [0.281–1.043]	0.067
COPD	2.345 [1.404–3.915]	0.001	0.846 [0.466–1.538]	0.584
QRS duration	0.975 [0.948–1.003]	0.080	*1.511 [1.160–1.959]*	0.026*
Hypertension	0.805 [0.469–1.380]	0.430	1.031 [0.570–1.863]	0.920
Dyslipidemia	2.619 [1.048–6.548]	0.039	1.232 [0.715–2.122]	0.452
Renal dysfunction	1.056 [0.454–2.456]	0.901	0.420 [0.012–1.531]	0.075
Hb1Ac	1.239 [0.760–2.020]	0.389	1.320 [0.021–3.426]	0.752
g. Multivariate cox regression analysis for parameters associated with AF events				
LVEF	0.964 [0.934–0.994]	0.021	*1.593 [1.122–1.986]*	0.006*
6MWT	1.001 [0.994–1.601]	0.977	1.024 [0.995–1.046]	0.712
BNP	1.020 [1.001–1.100]	0.008	*1.101 [1.001–1.210]*	0.017*
TNF alpha	1.180 [0.759–1.836]	0.462	1.298 [0.773–2.180]	0.324
GLP-1	1.049 [0.720–1.526]	0.804	1.087 [0.829–1.427]	0.962
CRP	1.010 [0.983–1.180]	0.561	0.997 [0.979–1.015]	0.732
IL-6	1.016 [0.808–1.277]	0.983	0.936 [0.703–1.247]	0.652
GLP-1 agonist	0.577 [0.348–0.957]	0.033	*0.603 [0.411–0.884]*	0.010*
Age	0.989 [0.964–1.014]	0.388	0.982 [0.957–1.008]	0.182
Obesity	0.941 [0.479–1.846]	0.859	0.952 [0.493–1.841]	0.885
NYHA 3	1.044 [0.756–1.441]	0.795	0.942 [0.670–1.324]	0.730
COPD	1.113 [0.785–1.577]	0.548	1.020 [0.683–1.523]	0.924
QRS duration	1.020 [1.003–1.037]	0.020	*1.182 [1.012–1.552]*	0.043*
Hypertension	0.929 [0.656–1.316]	0.680	1.519 [0.926–2.492]	0.098
Dyslipidemia	0.828 [0.560–1.223]	0.343	1.232 [0.715–2.122]	0.452
Renal dysfunction	0.588 [0.598–1.162]	0.126	0.613 [0.024–1.618]	0.082
Hb1Ac	1.176 [0.914–1.512]	0.208	1.150 [0.002–2.152]	0.073

GLP-1: glucagone like peptide 1; COPD: chronic obstructive pulmonary disease; NYHA: New York Heart Association; 6MWT: 6 min walking test; CRTd: cardiac resynchronization with a defibrillator; n.s.: not statistical significant; LVEF: left ventricle ejection fraction; ACE: angiotensin converting enzyme; ARS: angiotensin receptor; NOAC: new oral anticoagulant; DPP4: Di-Peptidil-Peptidasi IV; BNP: B type natriuretic peptide; CRP: C reactive protein; IL6: interleukine 6; TNFa: tumor necrosis factor alpha

p value < 0.05 is statistical significant, and marked as *

## Discussion

GLP-1 RA therapy in addition to standard hypoglycemic drugs induces at 6th and 12th month of follow up in CRTd patients an increase in the values of basal and post-prandial GLP-1. This is associated to other pleiotropic effects as the significant reduction of inflammatory biomarkers (CRP, IL6, TNFa), and of BNP values, as compared to patients under standard hypoglycemic drug therapy. At clinical level, GLP-1 RA treated patients vs. controls result in a significant amelioration of NYHA class, and of 6MWT. Consequently, CRTd patients in the GLP-1 RA groups vs. controls have a significant reduction of hospital admissions for HF worsening (without affecting the mortality) and of AF/VT events, and an increase in CRTd responders rate. In addition, we did not report statistical significant events of all cause deaths and cardiac deaths comparing patients in GLP-1 RA group vs. controls (p value > 0.05).

### 1. Biohumorals, hemodynamics, and anti-arrhythmics effects of GLP-1 RA therapy in addition to standard hypoglycemic drugs in HF patients with diabetes treated by CRTd

In CRTd patients with diabetes the GLP-1 RA therapy in addition to standard hypoglycemic drugs reduced the blood values of BNP at 6th and 12th month of follow up. Conversely, BNP blood values were predictive of hospitalization for HF worsening, and of AF events. BNP is a cardiac peptide relapsed in acute and chronic cardiac stress, and over stretching, and evaluated for the acute and chronic heart failure diagnosis [2, 10]. In fact, BNP measurements provide strong prognostic informations for all cause of deaths, and cardiovascular deaths in patients with heart failure [11]. However, higher BNP blood values are linked to advanced cardiac damage, and cardiac pump failure in patients with chronic HF [10–12]. Intriguingly, at last one of four patients undergoing CRTd will develop AF events [13]. However, a clear association may exist between AF episodes and BNP levels in CRTd patients. As first, the BNP is released from ventricular and atrial myocytes in response to cardiac wall stress [14]. Indeed, atrial hyper stretching and overload may contribute to elevate the synthesis and relapse of BNP [14], and this is consequently linked to higher rate of future AF events [14, 15]. In addition, in the long term all these adverse mechanical events shifting towards fibrotic processes, may lead to structural alterations of cardiac chambers in CRTd patients [16, 17]. Consequently, this may cause the failure of the cardiac pump in CRTd patients [16, 17], and in CRTd patients with diabetes [18, 19]. An index of the effectiveness of left ventricle pump is the LVEF, that is a parameter calculated by dividing the stroke volume by the end-diastolic volume [20]. However, in the DEFINITE trial the patients with greater improvement of LVEF at follow up experienced a significantly lower incidence of arrhythmic events [21]. On the contrary, a depressed LVEF was associated to worse clinical prognosis, as the result of a higher degree of anatomical ventricular remodeling, and pump failure in failing heart patients [22, 23]. Moreover, the advanced cardiac chambers dilatation and fibrosis may cause a severe depression of LVEF, that associated to the heterogeneity of cardiac repolarization properties, may contribute to an electro-anatomical remodeling [21]. To date, the electro-anatomical remodeling may persist also after the improvement of LVEF induced by CRTd, and it may cause a pro-arrhythmic status and a worse prognosis [21]. In this contest, a higher rate of arrhythmic events and worse prognosis may be seen in patients with longer duration of the QRS interval, as an index of right to left ventricle delay [24, 25]. In fact, the QRS duration is influenced by alterations in ionic channels conduction properties and by ventricular fibrosis, and scar extension, and it is a marker of inter-ventricular dyssynchrony [26]. Therefore, a higher degree of inter-ventricular dyssynchrony is linked to more pronounced electrical-anatomical alterations, and subsequently to higher arrhythmic burden, and worse prognosis in CRTd patients and in CRTd patients with diabetes [25–34]. In addition, we have to mention that, obese and non-obese patients with mild heart failure have a similar risk also of ventricular tachyarrhythmias, but that this clinical setting does not diminish the benefit of cardiac resynchronization therapy in these patients [35]. Nowadays, in a national cohort of patients eligible for CRTd, nearly 90% received a CRTd device, but unfortunately its use differed by race, implanting operator characteristics and hospital [36]. Moreover, in our study the GLP-1 RA group of patients experienced a reduction of the VT burden about a percentage of 4%, and of the AF burden about a percentage of 40%. In detail, we may speculate that, the T2DM may condition the cardiac ionic channels currents, affecting the depolarizing and repolarizing cardiac activity, and action potentials genesis, duration, and propagation in cardiac walls and heart chambers [9, 37]. In addition, in HFrEF patients with diabetes treated by CRTd, the concomitant anatomic remodeling related to cardiac fibrosis and scar disomogeneity and extension, may also contribute to the increase of the arrhythmic burden [4, 19, 38]. However, GLP-1 RA therapy in addition to standard hypoglycemic drugs may modulate these electrical properties [39], and this may lead to the reduction of the arrhythmic burden in CRTd patients with diabetes [4, 40].

### 2. Effects of GLP-1 RA therapy in addition to standard hypoglycemic drugs on clinical outcomes and CRTd responders rate in HF patients with diabetes treated by CRTd

GLP-1 RA therapy in addition to standard hypoglycemic drugs may reduce the endpoint of hospital admission for HF worsening in a percentage superior of 80%, and increasing with a 3.7 fold the CRTd responders rate in HF patients with diabetes. Numerous observations may explain these important study results. From literature data, the acute and chronic GLP-1 RA therapy in addition to standard HF therapy in NYHA class III/IV patients results in a significant improvement of LVEF, of peak oxygen consumption, of 6MWT, and of the quality of life score [39, 40]. To date, in our study we reported for first time in literature, that GLP-1 RA therapy in addition to standard hypoglycemic drugs vs. standard hypoglycemic drugs may reduce hospital admissions, without affecting mortality rate in CRTd failing heart patients with diabetes. Furthermore, patients in the GLP-1 RA group vs. controls experienced an increase in the CRTd responders rate. In fact, the Kaplan curve shows this effect at 12 months of up in GLP-1 RA group vs. controls, by a significant opening of the scissor at follow up end (p value < 0.05) (Fig. 4). The entity of CRTd response is based on clinical measures, as patients symptoms, and functional NYHA class assessment, and by echocardiographic diagnosis of LV reverse remodeling [2]. However, GLP-1 RA therapy in addition to standard hypoglycemic drugs, without affecting mortality may control the glycemic homeostasis in CRTd failing heart patients with diabetes, and by other pleiotropic effects may influence ionic channels properties and functions. This may result in the improvement of the electrical stability of cardiac membrane, and this may favor the genesis and propagation of cardiac potentials though the cardiac cells. All these effects may improve cardiac electrical, and hemodynamic functions also in patients with severe pump failure due to irreversible cardiac fibrosis. At clinical level, this may be translated in the amelioration of cardiovascular hemodynamic, associated to the reduction of the arrhythmic burden, HF symptoms, and NYHA class. Therefore, GLP-1 RA therapy in addition to standard hypoglycemic drugs may increase and enhance CRTd response, as a valuable and unique therapeutic effect vs. the conventional hypoglycemic drug treatments.

## Study limitations

All these study result have to be applied in a future study including a larger size of patients with diabetes, and at more long term follow up analysis. In fact, the small sample size and short duration of follow up may affect study outcomes. In addition, the present study shows data that are not supported by molecular experiments to assess the ionic and molecular alterations GLP-1 RA induced. To date, an animal model of heart failure under hyperglycemic stress condition may lead to the characterization of all these altered cardiac electrical and mechanical processes. In addition, we did not use a continuous monitoring systems for arrhythmias detection and devices interventions as described by authors [41], and this may affect the study outcomes. We may speculate that, all these pleiotropic and favorable electrical and hemodynamic effects GLP-1 RA induced may be translated in the future treatment of HF in CRTd patients with diabetes. However, further studies are needed to better understand the pleiotropic functions of GLP-1 RA therapy, and their cardiovascular effects. In future, a larger clinical trial may be adequate to assess all these pathogenic processes in a population of failing heart patients with diabetes treated by CRTd. This may be applied in clinical practice to reduce arrhythmic burden, hospitalizations, and to improve CRTd response in failing heart patients with diabetes.

## Conclusions

GLP-1 RA therapy in addition to standard hypoglycemic drugs vs. standard hypoglycemic drugs may significantly reduce inflammation, and BNP values in failing heart patients with diabetes treated by CRTd. These anti-inflammatory and hemodynamics effects are linked to significant improvement of LVEF, and to the reduction of the NYHA class, arrhythmic burden, and hospitalization for HF worsening. Intriguingly, GLP-1 RA therapy in addition to standard hypoglycemic drugs is associated to a 3.7 fold higher rate of CRTd responders vs. other conventional hypoglycemic drugs. Therefore, GLP-1 RA therapy in addition to standard hypoglycemic drugs may improve CRT responder rate and clinical outcomes in patients with diabetes. However, GLP-1 RA therapy in addition to standard hypoglycemic drugs may be recommended in T2DM failing heart patients treated by CRT-d.

## Abbreviations

AF: atrial fibrillation
BNP: B type natriuretic peptide
CRP: C reactive protein
CRT-d: cardiac resynchronization therapy with a defibrillator
DPP-4: dipeptidyl peptidase-4
ELISA: enzyme-linked immunosorbent assay
GLP-1: glucagon-like peptide 1
GLP-1 RA: glucagon-like peptide 1 receptor agonists
HF: heart failure
HFrEF: heart failure and reduced left ventricle ejection fraction
ICD: internal cardioverter defibrillator
IL6: interleukine 6
LV: left ventricle
LVEF: left ventricle ejection fraction
NYHA: New York Association Heart
T2DM: type 2 diabetes mellitus
TNFa: tumor necrosis factor alpha
VF: ventricular fibrillation
VT: ventricular tachycardia
